# Snail track ulcers of the tongue

**DOI:** 10.11604/pamj.2018.30.23.14118

**Published:** 2018-05-15

**Authors:** Andreia de Oliveira Alves, Fred Bernardes Filho

**Affiliations:** 1 Medical School, Centro Universitário Barão de Mauá, Ribeirão Preto, São Paulo, Brazil; 2 Dermatology Division, Department of Medical Clinics, Ribeirão Preto Medical School, University of São Paulo, Ribeirão Preto, Brazil

**Keywords:** Syphilis, treponema pallidum, tongue diseases

## Image in medicine

A 36-year-old male, heterosexual, complained of a 4-week history of ulceration in the tongue left margin. He denied previous treatment and any systemic diseases. The patient reported multiple sexual partners and episodes of unprotected sex in the past 12 months. Oral findings showed irregular erythematous patches on the dorsum of the tongue and erosive plaques on the tongue left margin with snail track pattern. On physical examination, cutaneous lesions were absent, and a diffuse micropolyadenomegaly was noted. The laboratory tests were positive for TPHA VDRL (1/64), and negative for HIV, hepatitis B and C. The diagnosis of snail track ulcers of secondary syphilis was performed. The patient was treated with two doses of intramuscular benzathine penicillin (total dose of 4,800,000 U IM) with an interval of seven days between each dose. The tongue lesions completely disappeared after the two doses of the penicillin. Although very rarely oral ulceration may be the only manifestation of infection. The two principal oral features of secondary syphilis are mucous patches and maculopapular lesions. The oral features of secondary syphilis can be painless or painful erythematous lesions, grayish-white mucous patches, or irregular linear erosions termed snail track ulcers. The resurgence of syphilis is mainly due to sexual risk behaviors. This cases illustrates the need for include syphilis in the differential diagnosis of oral ulceration and how clinical features should be valued in clinical semiotics.

**Figure 1:**
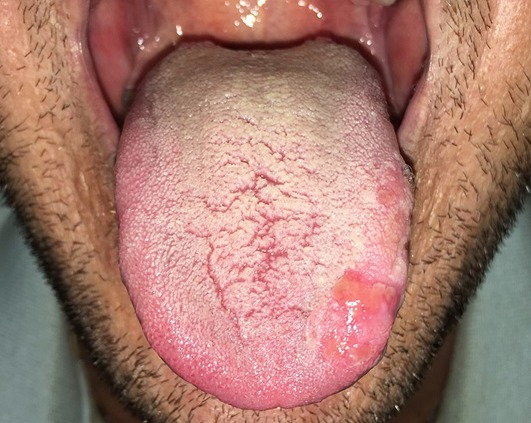
Erythematous whitish erosive plaques on the dorsum of the tongue with snail track pattern

